# Influence of water stress on engineering characteristics and oil content of sunflower seeds

**DOI:** 10.1038/s41598-022-16271-7

**Published:** 2022-07-20

**Authors:** Harby Mostafa, Mohamed T. Afify

**Affiliations:** Agricultural and Biosystems Engineering Department, Faculty of Agriculture, Benha University, Moshtohor, Qalyobia Egypt

**Keywords:** Plant sciences, Plant stress responses, Drought

## Abstract

Knowing some physical and mechanical characteristics and oil percentage of sunflower seeds could be useful for harvesting and processing equipment and activities such as transportation, storage, food production processes and establishing database of this seed. The main aim of this research was to study the effect of water stress during irrigation on seed’s properties and quality. For this purpose, a field experiment was done under four deficit irrigation treatments [80%, 60%, 100–80 (100% irrigation requirement ETc to seed formation, and then reduced to 80% until harvesting) and 100–60% (100% ETc to seed formation, and then reduced to 60% until harvesting)] in comparable with full irrigation (100%). Geometrical, gravimetrical and mechanical characteristics as well as oil seed content and yield of sunflower seed were estimated. Result showed that there was no significant effect of low (100–80%) and medium (80%) irrigation deficit treatments on geometrical, gravimetrical and mechanical characteristics, while applying 60% of irrigation requirement (ETc) showed a significant effect on them. On the other hand, low and medium irrigation stress treatments improved the oil yield and seed oil content. The highest increase was 8.54% and 5.6% for oil yield and oil content respectively, considering T_100–80_ followed by applying 80% ETc, but with high water stress (60% ETc) oil yield and seed oil content significantly decreased.

## Introduction

Water is important in agricultural production, however in the near future, a water scarcity will have to be addressed. In semi-arid Mediterranean regions, water scarcity and rising rivalry for water resources between horticulture and other sectors compel the use of water management strategies that allow for water conservation while maintaining acceptable levels of production^1,2^.

Since sunflower has the capability to survive under stress condition than other oilseed crops to some extent, also water deficit in combination with high air temperature from flowering to grain filling stages significantly reduced the seed yield and seed oil quality of sunflower in the arid and semi-arid region^3^. Deficit irrigation is one of methods for increasing water use efficiency. Applying water deficit (80%)^4^ produced almost the same yield of sunflower seeds than that obtained from full irrigation, besides saving about 20% of irrigation water and maximized water use efficiency. Biological yield of safflower genotypes significantly reduced in drought stress by 17.9%, compared to normal condition^5^. Oil percentage is an important criterion for determining sunflower quality, and it can be influenced by irrigation deficits^6^. The oil content of sunflower seeds ranges from 37 to 42%^4^. According to prior research, when the sunflower crop was subjected to water stress during the flowering stage, the percentage of sunflower oil content reduced dramatically^7^. A decrease in safflower oil content with rising in drought has been reported by^5^ where the results showed a significant decrease of oil yield in drought stress conditions by 19.3%. In most of the cases oil yield reduction is less than seed yield reduction which indicates increase in oil contents. But severe drought at flowering and bud stage reduced oil yield more than seed yield which may be due to decrease in seed oil contents^3^. So these two stages may be regarded as the most sensitive to drought stress.

Because of the need of conserving and supplying water, many crops are now using tiny irrigation systems. Experiments have revealed that some plants respond positively to yield and its qualities, while others do not^8^.

Scientists are researching the physical, mechanical, chemical, and botanical aspects of seed from an engineering standpoint in order to improve seed production while maintaining high quality. These characteristics can be used to drive the design of seed production equipment and processes, such as planting, harvesting, processing, and testing specific cleaning and sorting machinery^9–11^. For instance, the size and shape of seeds are important for either their electrostatic separation from undesirable materials^12^. Also, the identification of seed shape could be important for an analytical prediction of its drying behavior^13^. Bulk density, true density and porosity are useful in sizing grain hoppers and storage facilities as they can affect the rate of heat and mass transfer during aeration and drying operations^14^. Data on the physical qualities of agro-food materials are significant because they may be used to input into models that anticipate product quality and behavior in sowing, handling, pre-harvesting, and post-harvesting circumstances; and they can help understand how food is processed^15^.

Shape and size, length, width, thickness, volume, geometric diameter, mathematical diameter, percentage of sphericity, flat surface area, transverse surface area of individual seeds, stiffness force, resting angle, friction coefficient, terminal velocity of threshed head components, drag coefficient, real density, and bulk density are the most important properties affecting sunflower seeds. In addition, the width of the head, the number of seeds per head, and the weight of 1000 seeds are the most essential properties affecting the head^16^.

The results of physical and mechanical properties of sunflower seeds showed a variation of 14.32 to 31.00 mm for length, 4.7 to 9.8 mm for width and 2.7 to 6.6 mm for thickness of sunflower seeds. The values of 1000 seed mass, volume, true density, bulk density and porosity were between 149.8–167.7 g, 99.05–628.9 mm^3^, 444.5–521.8 kg/m^3^, 269.06–275.57 kg/m^3^ and 39.09–47.18% respectively. The rupture force, deformation, and absorbed energy increased with increase in moisture content from 1.8 to 14.5%, while decreased with further increasing of moisture content from 14.5 to 20.3%. The mean value of percentage of physically damaged seeds increased from 2.75 to 10.81% with increasing the impact velocity from 40.8 to 62.3 m/s. In both impact orientations, the total damaged seeds increased with increase in impact velocity for all moisture contents of seeds^12,17–19^.

There has been research on the engineering properties of sunflower seeds^15,16,20^, but little is known about how agricultural techniques affect engineering properties such as water stress. Irrigation and planting systems might also have an impact on seed properties^4^.

The goal of this research is to find out how using water stress systems influences sunflower plant engineering features, productivity, and seed oil yield. So, if some deficit irrigation treatments have a positive effect on productivity and seed oil yield, then an amount of water can be saved.

## Materials and methods

To meet the goals of this study, a field experiment utilizing the sunflower hybrid Sakha 53 was undertaken at a private farm in Qalyubia Governorate, Egypt, during two successeve growing summer seasons of 2019–2020 (Permissions were obtained from Egyptian Ministry of Agriculture and experimental research and field study was comply with relevant institutional, national, and international guidelines and legislation). This site exemplifies the Nile Delta's clay soil conditions. Sunflowers have a growing seasons that lasts from June to September. Throughout the profile, the dominating soil at the experimental location was clay-textured (2.45% coarse sand, 18.55% fine sand, 27.77% silt and 51.23% clay). The region is arid characterized, during that time of year, by no rainfall, a high average temperature and a relatively medium humidity resulting in a medium to high evaporative demand.

The experimental site was divided to five plots (one for each irrigation treatment) have a width of 3 m and extend over a length of 25 m with 2 m separation line between plots. Seeds were planted at 30 cm between plants with spacing 60 cm between rows, that is, each plot is divided into five rows, and each row is considered a replication.

For irrigation was used polyethylene (PE) laterals of 16 mm diameter with 4 L/h built-in drippers at 30 cm apart were used with one lateral for each row. The control valves were installed at the inlet of each treatment to control the flow of water. One bar pressure was maintained using 0.75 kW pump.

The treatments for irrigating the sunflower crop were full irrigation at 100% of crop requirement (ETc) and four water deficit regimes [80% ETc, 60% ETc, (100–80% ETc) and (100–60 ETc) denoted as T_100_, T_80_, T_60_, T_100–80_, and T_100–60_, respectively]. The T_100–80_ and T_100–60_ treatments were applied as 100% ET_C_ to seed formation and then reduced to 80% and 60% ETc until harvesting, respectively.

Values of daily evapotranspiration (ETo) were obtained from data predicted by Central Laboratory for Agricultural Climate (CLAC) which are always available 5 days beforehand. Kc for sunflower during the growing season was obtained from FAO (2015). The obtained ETo and Kc were used to calculate water requirement for sunflower ETc (mm) as described by^21^.

The irrigation starts on all treatments when 75% of the available water under 100% ETc irrigation treatment is consumed. All other agricultural practical for sunflower crop was used as recommended by the Agriculture Ministry.

At harvest time, the heads of 15 plants were randomly drawn from each plot and were separately harvested, bagged, and dried under the sunshine for one week. Half of harvested seeds used for measuring oil yield and the other half used for measuring seed physical and mechanical characteristics.

Sunflower oil yield per unit area is result of seed yield per unit area and the oil percentage of seed. Using the Soxhlet equipment and petroleum ether 40–60 °C as a solvent, the seed oil percentage was measured. The powdered samples were soaked in n-hexane 64–68 °C for 48 h with periodic shaking after being air-dried samples of sunflower seeds were milled twice in a stainless-steel experimental mill. The food was immersed in the same solvent for a second time for another 24 h. The mixed extracts were filtered through sufficient amounts of Anhydrous Sodium Sulphate and then vacuum-distilled to remove the solvent^22^.

To obtain the moisture content, 50 g of seeds for each treatment were selected and placed for 24 h at 72 °C in the oven and in accordance with ASAE Standard^23^ moisture was measured on the basis of dryness (d.b).

Mass of three groups of 1000 seeds for each sample was measured using a balance with an accuracy of 0.001 g. The seeds size were measured for three groups of 100 seeds by a caliper with accuracy of 0.01 mm. Because the shape of the seeds is irregular granular, seed size is expressed by geometrical diameter (dg) as following^20^:

$$ d_{g} = \left( {TWL} \right)^{1/3} $$

where: T, W and L are the thickness, width and length of the seed. In addition, surface area (S), volume (Vs) and sphericity coefficient (Ø) of seeds, were determined using following equation^17^:

$$ S = \pi \times d_{g}^{2} $$

$$ V_{s} = \frac{{\pi d_{g}^{3} }}{6} $$

$$ \phi = \frac{{d_{g} }}{L} $$

Filling a container with 500 mL of seeds from a height of 15 cm, hitting the top edge, and measuring the contents, the bulk density (Pb) was estimated 3 times for each sample. The true density (Pt) of a substance is defined as the ratio of its mass to its actual volume. Liquid displacement method was used to determine true density, and toluene fluid (C7H8) was used due to the low absorption of liquid by the seed. A certain mass of seed was poured into a cylindrical container having a volume of 100 mL. Then, the volume of transferred toluene was recorded, and the true density of the seeds was determined using the mass ratio of the seed to the displaced liquid volume^24^. The porosity (Pf) of bulk seeds is defined as the proportion of space that is not occupied by the seeds. The percentage of porosity was calculated using the formula below^12^.

$$ P_{{\text{f}}} = \left( {1 - \frac{{\rho_{{\text{b}}} }}{{\rho_{{\text{t}}} }}} \right) \cdot 100 $$

The crushing load plays a very important role in the design of loading and unloading equipment, storage systems, harvesting machines and drying equipment, conveyors, spouting and free falling equipment, because seeds are affected by other metallic, wood and plastic surfaces during operations in this equipment, which can result in mechanical damage^13^, whereas oil expulsion could be used for rupture force and rupture energy^17^.

**Figure 1:**
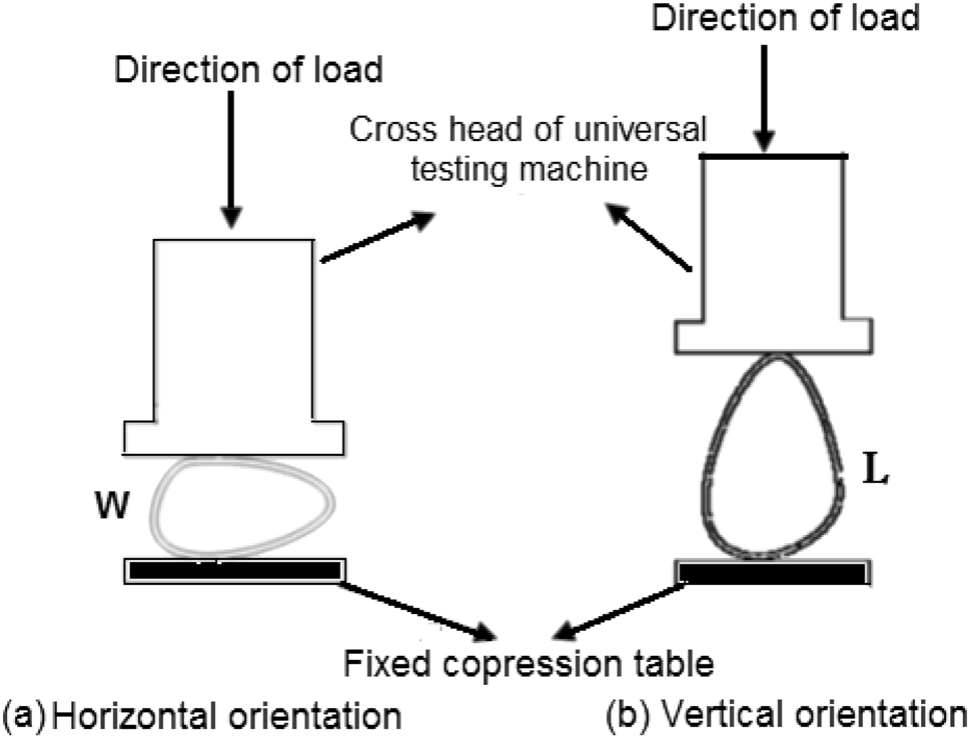
Crushing load testing machine as described by Khodabakhshian et al.^14^.

To assess the impact of the loading direction on the rupture, the seeds were placed vertically, with the seed's main axis in line with the loading direction, and horizontally, with the main axis perpendicular to the loading direction, using the Instron Universal Testing Machine (Fig. 1)^14^.

Using the emptying approach, the seeds’ repose angle (θ) was calculated in a bottomless cylinder (diameter 5 cm; height 10 cm). The cylinder was filled with sunflower seeds and gradually raised until a heap was produced on a table with three distinct surfaces (wood, stainless steel, and plastic). The heap's diameter (D) and height (H) were measured and repose angle (θ) was calculated as reported by^25^:

$$ \theta = \tan^{ - 1} \frac{2H}{D} $$

Static friction coefficients (μ) on the three different surfaces (wood, stainless steel, and plastic) were calculated for seed. These surfaces are widely used in seed processing and handling^25^. Static friction coefficients were calculated as a tangent of slope angle (angle of repose)^26^:

$$ \mu = \tan \theta $$

## Results and discussion

### Geometrical, gravimetrical and mechanical characteristics of sunflower seed

The variation of the length, width, thickness, and moisture content, of the sunflower seed at different irrigation treatmens are shown in Tables 1 and 2. The results showed that all these parameters decreased with increasing the water deficit. The moisture content of seeds was affected by water stress, where it decreased from 7.92% at T_100_ to 5.49% at T_60_. All dimensions have a similar tendency. The average length, width, and thickness decreased by 14%, 7.6%, and 7.1%, respectively. In addition, the 1000 seed mass decreased by 9.8% with applying the T_60_ treatment compared to the T_100_ treatment. The geometric mean diameter, volume, and surface area had the same behavior as shown in Table 2. These behaviors may be due to the complexity of water stress impacts on seed moisture content as reported by^17,27^. The reduction trend in dimensions with seeds' moisture content was due to the filling of capillaries and voids upon absorption of moisture and subsequent swelling as described by^15^. The variation of the length, width, thickness, geometric mean diameter and sphericity, of the sunflower seed increased with increasing moisture content from 3 to 14% d.b for all size categories. This indicates that during the moisture absorption process, the sunflower seed will simultaneously expand in all dimensions as reported by^14,19^.

**Table 1:** The dimensions, mass and moisture content for seeds.

	Length (mm)	Width (mm)	Thickness (mm)	1000 seed mass (g)	Moisture content (%)
**T**_**100**_					
Mean	12.89	6.58	4.36	89.89	7.92
SD	0.63	0.52	0.38	2.11	0.21
CV	5.39	7.94	8.81	5.32	1.33
**T**_**80**_					
Mean	12.81	6.54	4.39	89.71	7.63
SD	0.69	0.41	0.31	2.30	0.29
CV	5.14	6.25	7.60	5.76	2.31
**T**_**60**_					
Mean	11.08	6.08	4.05	81.07	5.49
SD	0.53	0.34	0.27	3.92	0.33
CV	4.40	5.32	6.49	6.87	2.40
**T**_**100–80**_					
Mean	12.37	6.54	4.32	89.03	7.52
SD	0.44	0.53	0.31	2.21	0.19
CV	3.58	7.69	7.65	6.01	2.03
**T**_**100–60**_					
Mean	12.34	6.48	4.28	86.19	6.47
SD	1.18	0.42	0.38	2.41	0.25
CV	9.46	6.43	8.43	5.72	1.77

*SD* standard deviation, *CV* coefficient of variation %.

**Table 2:** Geometrical and gravimetrical properties of sunflower seeds at different water treatments.

	d_g_ mm	S mm^2^	V_s_ mm^3^	Ø	*p*_b_ kg/m^3^	*p*_t\_ kg/m^3^	*P*_*f*_ %
**T**_**100**_							
Mean	6.93	151.15	175.50	0.59	487.1	829.5	41.67
SD	0.33	14.08	24.16	0.04	30.11	44.3	2.21
CV	4.75	9.32	13.76	6.82	12.14	15.87	6.11
**T**_**80**_							
Mean	6.90	150.50	174.49	0.57	485.9	829.2	41.60
SD	0.26	11.69	20.70	0.03	26.23	53.74	3.23
CV	3.77	7.61	11.54	4.98	10.21	23.07	7.65
**T**_**60**_							
Mean	6.26	138.01	161.75	0.58	440.1	711.3	38.13
SD	0.21	9.00	15.44	0.03	23.49	48.94	4.05
CV	3.05	6.08	9.10	4.34	14.43	19.44	11.21
**T**_**100–80**_							
Mean	6.90	150.54	175.47	0.56	487.9	839.7	41.90
SD	0.26	11.39	19.79	0.03	28.33	61.65	3.02
CV	3.80	7.62	11.47	4.84	9.55	32.10	7.88
**T**_**100–60**_							
Mean	6.85	149.24	170.09	0.57	448.2	760.0	41.03
SD	0.41	18.35	32.97	0.04	20.34	51.66	2.87
CV	5.74	11.52	17.35	6.35	11.03	29.50	8.34

The geometrical diameter (d_g_), surface area (S), volume (V_s_), sphericity coefficient (Ø), bulk density (Pb), true density (Pt) and porosity (Pf).

The majority of widths are about 1.5 thicknesses, and the elongation indicates that sunflower seeds have a low oblong shape. Sunflower seeds, on the other hand, are more likely to roll than slide due to their medium elongation ratio. This was also revealed by the sphericity data in Table 2. This information could be useful in the design of separators, and conveyer equipment. The mean values of sphericity of the sunflower seeds used in this study were much higher than those reported by^18^, while the sphericity values for the sunflower seeds were in the same ranges compared with those of this study^28^. The calculated porosity decreased from 41.6% (T_100_) to 38.1% (T_60_) when moisture content decreased from 7.9 to 5.5% (w.b.) that affected by water stress. The form of the plot was similar to those were observed by^20^. Also, the geometrical diameter (d_g_), surface area (S) and volume (V_s_) were take the same trend.

Coefficients of static friction of seed on studied surfaces increased from 0.380 to 0.424 as water stress increased from T_100_ to T_60_ as shown in Table 3. This may be explained by increased cohesive force of seeds with the surface because of the dimensions shrank that happened as result of water stress. The results showed that the highest value of static coefficient of friction was on the wood surface, followed by plastic, and stainless steel surfaces at the same water stress for all treatments. In addition, the higher coefficients for high water stress might be attributed to its lower sphericity of shape compared with that of full irrigated (T_100_). The variation of the angle of repose take the same trend, where it increased by 9.5% (stainless steel), 4.3% (plastic) and 4% (wood) as water stress increased from T_100_ to T_60_. The angle of repose increased linearly with an increase in the water stress because seeds might stick together which results in less flowability and better stability, thereby increasing the angle of repose. Similar findings were reported for sunflower seed and kernel^14,17^, and sesame^19^.

**Table 3:** Repose angle and coefficients of static friction of sunflower seeds at different surfaces and water treatments.

Treatments	Repose angle (degrees)			Coefficients of static friction		
	Stainless steel	Plastic	Wood	Stainless steel	Plastic	Wood
T_100_	21	23	25	0.380	0.424	0.466
T_80_	22	24	25	0.410	0.445	0.466
T_60_	23	24	26	0.424	0.445	0.488
T_100–80_	23	24	24	0.424	0.445	0.445
T_100–60_	22	23	24	0.410	0.424	0.445

The obtained results of crushing load (Table 4) shows that the greater values were in the vertical direction than horizontal direction for all investigated treatments. The maximum crushing load reaches 63.1 and 25.0 N for T_100_, while the minimum value was 46.2 and 21.4 N for T_60_ for both vertical and horizontal directions respectively.

**Table 4:** Effect of water stress and orientation of loading on crushing load (N).

Treatments	Mean	SD	CV
**T**_**100**_			
Vertical	63.1	3.78	6
Horizontal	25.0	2	8
**T**_**80**_			
Vertical	59.5	4.03	6.78
Horizontal	23.6	1.78	7.53
**T**_**60**_			
Vertical	46.2	3.52	7.62
Horizontal	21.4	4.3	20.09
**T**_**100–80**_			
Vertical	61.7	3.65	5.92
Horizontal	24.6	1.9	7.71
**T**_**100–60**_			
Vertical	55.6	4.03	7.25
Horizontal	22.5	3.44	15.29

The crushing load increases as the seed size increases, maybe because the seed contact area with the loading plates expands, resulting in the expansion of low stress. This is consistent with Hertz's compression test theory for food items^14^. Both orientations exhibit the same trend of increased crushing load as the moisture content rises from 5.49% (T_60_) to 7.92% (T_100_). These findings are consistent with those of^20^, who discovered that raising the moisture content of sunflower seed from 3 to 8% d.b increases the crushing load. This could be explained by the gradual change in the integrity of the cellular matrix or cellular structure of seeds^19^.

### Oil seed content (%) and yield

When full irrigation was applied to seed formation and then reduced to 80% ETc until harvesting (Table 5), the highest oil percentage (40.32%) was recorded, followed by T_80_ for the entire growing season (39.67%), and the lowest percentage (36.12%) when plants were subjected to water stress T_60_. There was no substantial effect on oil seed content when water stress occurred after the seed filling stage^29^. The decrease in oil percentage in the control treatment (T_100_) could be due to increased water consumption, which leads to excessive vegetative growth and delayed maturation of immature seeds at harvest time. The decrease in oil percentage in the severe stress treatment could be due to impaired seed filling, which causes the skin of sunflower seeds to thicken^27,30^. It has been quoted that the oil percentage does not damage in low water stress^4^.

**Table 5:** Effect of water stress on seed oil content and yield of sunflower.

Parameter	Water treatments					LSD_0.05_
	T_100_	T_80_	T_60_	T_100–80_	T_100–60_	
Oil (%)	38.18^bc^	39.67^ab^	36.12^d^	40.32^a^	37.32^cd^	1.54
Oil yield (Mg/ha)	1.487^b^	1.503^b^	1.359^c^	1.614^a^	1.581^ab^	0.108

Means followed by the same letter are not significantly different from one another based on LSD at P ≤ 0.05.

The intensity of oil yield change depends on the growth stage of the crop and the percentage of water reduction. The oil yield of sunflower was affected by drought stress, with the low status treatment yielding 8.6% for T_60_ less than the control treatment, while oil yield was increased by 8.5% for T_100–80_. Water stress during the flowering stage was found to be a limiting factor for seed filling, resulting in a significant reduction in oil yield^31,32^. The effect of water scarcity on seed oil yield also emphasizes the importance of attention to water stress potential in different sunflower genotypes.

## Conclusion

The physical and mechanical properties are important to be known for post-harvest technology, and deficit irrigation has to be used as effective method for water saving. Comparing the irrigation treatments from a physical and mechanical properties point of view, all physical and mechanical parameters studied in this research were close to each other under all treatments except under 60% ETc treatment when all parameters were decreased significantly. The repose angle increased from 21° to 26° whereas, the coefficient of static friction varied from 0.380 to 0.488 over different material surfaces in the specified water stress. On the other hand, low and medium irrigation deficit treatments improved the oil yield and its oil content. The highest increase for oil yield and seed oil content were under applying T_100–80_ (100% ETc to seed formation, and then reduced to 80% until harvesting) followed by applying 80% ETc, but with high water deficit (60% ETc) oil yield and seed oil content significantly (P ≤ 0.05) decreased.

## Data Availability

All data generated or analysed during this study are included in this published article.
